# Fathers Matter Too: Investigating Their Role with the P-CRS

**DOI:** 10.3390/pediatric17020038

**Published:** 2025-03-18

**Authors:** Alexandro Fortunato, Maria Quintigliano, Costanza Franchini, Marco Lauriola, Anna Maria Speranza

**Affiliations:** 1 Department of Dynamic, Clinical Psychology and Health, Faculty of Medicine and Psychology, Sapienza University of Rome, 00185 Roma, Italy; alexandro.fortunato@uniroma1.it (A.F.); costanza.franchini@uniroma1.it (C.F.); 2 Department of Systems Medicine, University of Rome Tor Vergata, 00133 Rome, Italy; maria.quintigliano@uniroma2.it; 3 Department of Social and Developmental Psychology, Sapienza University of Rome, 00185 Rome, Italy; marco.lauriola@uniroma1.it

**Keywords:** father–child relationship, parent–child relationship scale (P-CRS), relationship assessment, infancy, early childhood

## Abstract

Background: The development of children is shaped by a complex interplay of biological, psychological, and social factors, yet the role of fathers remains underrepresented in research. This study contributes to addressing this gap by examining paternal involvement through the Parent–Child Relationship Scale (P-CRS), an observational tool previously validated for mother–child interactions. Methods: The sample included 204 father–child dyads, with children aged 6 months to 5 years (mean age: 43.3 months), encompassing both clinical and non-clinical groups. Experienced clinicians conducted in vivo observations across 4–5 sessions, scoring interactions along three dimensions: parent, child, and interaction. Results: Confirmatory factor analysis (CFA) validated the P-CRS’s applicability to father–child relationships, confirming its psychometric robustness and alignment with the factors observed in mother–child dyads. Conclusions: These findings highlight the unique and complementary role of fathers in shaping developmental trajectories and underscore the importance of incorporating fathers in parenting interventions and assessments. Additionally, they demonstrate the P-CRS’s effectiveness in capturing the nuanced dynamics of early parent–child relationships. Future research should investigate longitudinal differences in parental roles and expand the P-CRS’s application to diverse family structures.

## 1. Introduction

Assessing parent–child relationships during infancy and early childhood is a crucial tool for understanding children’s development, both in its adaptive and maladaptive aspects. As widely recognized, children’s difficulties at this developmental stage are deeply rooted in their early interactions with caregivers and may reflect the presence of relational disorders [1]. The level of flexibility, mutual regulation, and contingency within these interactions offers valuable insights into the quality of the relationship, helping to determine whether children’s difficulties are temporary or indicative of a more persistent disorder, as well as how the relationship itself is affected by the ongoing condition.

For each child, specific developmental trajectories are shaped by the interplay of various factors, with risk and protective factors playing a central role [2]. Early relationships themselves can function as either risk or protective factors, depending on their quality [3,4], highlighting the importance of evaluating these relationships during infancy and early childhood to better understand children’s developmental pathways. To do that, it is crucial to identify the various aspects of these relationships.

As highlighted by the Mutual Regulation Model [5], the bidirectional influence between children and their caregivers, beginning in the earliest stages of development, necessitates an assessment that encompasses not only the specific traits and characteristics of both members of the dyad (e.g., the degree of a parent’s intrusiveness or child’s withdrawal within the interactions), but also the overall quality of their relationship (e.g., the ability to manage stressful moments, adapt flexibly to situations, and restore a sense of pleasure after conflict).

This aspect is of primary interest to clinicians working with infants and children, as it provides insights into the origins of specific developmental challenges and enables preventive interventions [6]. To this end, evaluating early relationships using a variety of tools offers valuable guidance to clinicians by enhancing the diagnostic process with critical information. Observational tools may play a key role in this process by ensuring that the information provided by parents is complemented by the direct assessments of clinicians.

The observation of parent–child interactions by a clinician, along with the use of observational rating scales, are pivotal aspects of the diagnostic process in infancy and early childhood. These methods enable the identification of relational dynamics and child functioning characteristics that parents may not fully recognize or be able to report through self-report questionnaires.

It is precisely because of the central role that the parent–child relationship plays in development that major diagnostic manuals for infancy and early childhood, such as the Diagnostic Classification of Mental Health and Developmental Disorders of Infancy and Early Childhood (Revised Edition) (DC:0–3R; [7]), DC:0–5 [8], and the Psychodynamic Diagnostic Manual (PDM-2; [9]), have developed evaluation systems and provided significant guidance on how to assess it. These diagnostic systems place the quality of early relationships at the center of the evaluation, focusing on the intensity, frequency, and duration of maladaptive interactions. This approach enables the assessment of whether relationships are well adapted or impaired and, consequently, whether they are characterized by the presence of relational disorders.

Within this theoretical and clinical framework, the Parent–Child Relationship Scale (P-CRS; [4]) was developed to evaluate early relationships. It is an innovative observational tool that not only evaluates the overall quality of the parent–child relationship through common interactive patterns but also identifies the specific contributions of both members of the dyad. Developed from the multiaxial diagnostic model of DC:0–5 [8] and PDM-2 [9], the P-CRS has proven effective in distinguishing between clinical and non-clinical conditions, as well as identifying specific risk factors for child development.

Speranza et al. [4] observed and evaluated interactions within mother–child dyads, identifying specific dimensions of the relationship based on the contributions of both the child and the parent, as well as the overall quality of their interaction. Moreover, they demonstrated that typical interactive patterns were associated with distinct diagnostic conditions in children. For example, children with affective and relational disorders exhibited more conflict, rigid interaction patterns, and anxiety in their relationships with their mothers.

Quintigliano et al. [10] compared clinical and non-clinical mother–child dyads to assess whether the P-CRS could differentiate between clinical and non-clinical conditions and capture specific risk factors for the development of affective and relational disorders in children. While the authors emphasized that the scale is not intended as a diagnostic tool, they also highlighted its value in identifying specific risk factors associated with certain clinical conditions. For instance, scores exceeding the cutoff for the “withdrawn child” scale were linked to autism spectrum disorder (ASD).

Studies on the P-CRS, as well as on the parent–child relationship in general, have primarily focused on the mother–child relationship (i.e., [11,12,13,14,15]). This is partly due to theoretical gaps, the often-limited willingness of fathers to participate in such studies, and the lower social recognition of their impact on child development [16]. However, research is increasingly exploring the role of fathers in their relationships with their children and their impact on child development, producing some noteworthy findings [17]. Indeed, since the 1990s, a combination of theoretical advancements, policy interventions, and social transformations has led to a surge in research on fatherhood, emphasizing the importance of paternal involvement in child development [18]. This shift introduced a new paradigm in the understanding of fatherhood, driven largely by the conceptualization of Lamb et al. [19], who argued that paternal commitment encompasses three key dimensions: engagement, accessibility, and responsibility. This framework has significantly influenced subsequent research [18]. For instance, early studies reported that positive paternal involvement was associated with enhanced cognitive and emotional development, as well as improved academic performance and behavioral outcomes in children [20,21].

Some studies have adapted tools commonly used to analyze the mother–child relationship to the father–child relationship, finding both many similarities and some differences such as in the domain of playfulness (i.e., [22,23]). Additionally, various psychological constructs, such as reflective function and attachment, have been examined in fathers as well, showing that like mothers, they impact the child’s development [24,25]. Parental sensitivity, for instance, has shown a certain assimilation between maternal and paternal sensitivity [26], as well as the consequences for the child’s behavior.

Regarding the impact of fathers on child development, there are different viewpoints: some support the idea that the contributions of mothers and fathers are largely overlapping, while others emphasize their uniqueness. However, it is now recognized that the constructs related to parenting apply to both fathers and mothers and are not exclusive to one gender or the other. Fathers’ behaviors influence the development of children just as much as mothers’ behaviors do, and differences in parental roles are gradually diminishing, particularly in relation to caregiving, time spent with children, and nurturing behaviors [27].

Moreover, family structures have changed over the past few decades, leading to a significant increase in families with same-sex couples (whether fathers or mothers). Emerging research on same-sex parent families indicates that parenting abilities and their impact on development are not defined by gender (e.g., [28,29]). Consequently, it is essential to use measures that go beyond a sole focus on mothers in caregiving roles and that can effectively assess the relational dynamics between parents and children in these families.

The literature widely agrees that children’s adjustment is influenced more by parenting quality than by the parents’ gender [30]; however, it is, nonetheless, essential to understand the unique contributions that parents can make to their relationship with their children in order to further identify the factors that promote their adjustment.

It should be emphasized, however, that while both parents influence child development positively and negatively, this does not mean that their contributions are identical. At the same time, children’s contributions may also vary depending on the parent they are interacting with. Rather, fathers and mothers contribute in unique and complementary ways to the child’s development, without a hierarchical relationship between their roles [16,31,32,33]. Regarding the differences highlighted in the literature between maternal and paternal roles, mothers are generally more nurturing and emotionally supportive, while fathers tend to focus more on preparing children for future challenges and offer a long-term perspective on their behavioral patterns. This is particularly relevant to the child’s age: the younger the child, the greater the need for care, often provided by the mother. However, it is noteworthy that fathers, through their ability to perceive and address their children’s unique needs, can help them avoid difficulties in life. Furthermore, regarding gender, fathers seem capable of providing greater emotional support to daughters, sometimes equal to or even surpassing that offered by mothers [16]. Paternal involvement in relationships with preschool-aged children has also been associated with a reduced risk of developing internalizing and externalizing disorders [34].

Fathers appear to contribute uniquely to their children’s development, particularly in relation to emotional regulation, risk assessment, and cognitive development through stimulating play. However, the literature on the role of father–child relationships requires a deeper understanding of how these interactions influence children’s development, having long been constrained by measurement tools largely based on models focused on mothers. As highlighted by Cabrera et al. [35], it is essential to develop assessment tools capable of capturing the specific patterns of interaction between fathers and their children and how these affect their development.

Based on these considerations, this study aims to apply the P-CRS [4] to a group of fathers and conduct a confirmatory factor analysis on this group based on the exploratory factor analysis previously conducted on mothers [4]. While acknowledging the specificities and potential differences between the father–child and mother–child relationships, we hypothesize that the same dimensions identified with mothers can be confirmed. This is because we are referring to parental relational dimensions that should not be influenced by the parent’s gender or family role, but rather by the quality of parenting, the interaction, and the child’s contribution.

## 2. Materials and Methods

### 2.1. Participants and Procedure

The sample included 204 father–child dyads (see Table 1). The children were categorized based on the following conditions: 36.8% had no clinical condition (N = 75); 10.3% had a neurological disease, organic illness, or genetic condition (N = 21); 13.7% were diagnosed with autism spectrum disorder (N = 28); 19.1% had developmental delay (N = 39); 8.3% were born prematurely (N = 17); 6.9% had affective or relational disorders (N = 14); and 4.9% had a feeding disorder (N = 10). It is important to note that the inclusion criteria excluded overlapping characteristics between groups (e.g., children with developmental delay who were also born prematurely were not included in both groups). If children met the criteria for both diagnoses, they were excluded from the study to ensure clear group distinctions and avoid overlap.

The fathers had a mean age of 38.4 years (SD = 5.9), while the children ranged in age from 6 months to 5 years, with a mean age of 43.3 months (SD = 16.5). The sample included 127 male and 77 female children. There were no significant differences in the distribution of fathers’ and children’s ages across the six clinical and non-clinical groups. Most of the children were Italian (93.6%), as were the fathers, who were either separated or divorced (57.8%) and employed (99.5%).

The dyads were observed by experienced clinicians with at least 10 years of clinical practice (for details, see [4,10]). These clinicians, selected randomly, are psychologists, psychotherapists, and psychiatrists specializing in developmental age. Clinicians completed the P-CRS (Parent–Child Relationship Scale) after observing each dyad during 4–5 sessions. Each dyad was observed and evaluated by a single clinician. Observations were scored using a 5-point Likert scale (1 = not at all descriptive; 5 = strongly descriptive). Clinicians were informed about the importance of their collaboration, trained by a member of the research team, and supervised during the study. The training involved observing and applying the instrument. During the training, the clinicians conducted reliability tests on test cases, and only after achieving good reliability were they allowed to apply the scale. The recruited clinicians applied the observation to the cases they were actively following, who voluntarily decided to participate. The clinicians worked in various public and private centers specializing in developmental mental health. Observations were conducted during routine clinical visits, typically scheduled weekly, and did not require a specific setting. Instead, dyads composed of fathers and children without a diagnosis were recruited through schools. Families who voluntarily chose to participate took part in observations and evaluations conducted by experienced clinicians in the rooms of the university department that led the research. Sessions, lasting about one hour, often involved play or free interaction. Although the P-CRS is not strictly an observational measure, it serves as a tool for clinicians to guide their evaluations during and after observations to inform the final assessment. As such, the use of the P-CRS is independent of specific procedural or theoretical orientations. All the observations were conducted in vivo by the clinicians. The participants were informed about the study by the rating clinicians, who guaranteed anonymity and confidentiality. Written informed consent was obtained from all the fathers included in the study. The study was approved by the Ethical Committee of the Department of Dynamic and Clinical Psychology, Faculty of Medicine and Psychology, Sapienza University of Rome. All the procedures adhered to the ethical standards of institutional and national research committees, as well as the 1964 Helsinki Declaration and its later amendments or comparable ethical guidelines.

### 2.2. Measures

The Parent–Child Relationship Scale (P-CRS; [4]) is a clinician-report tool that evaluates the relationship between parents and their children in early childhood or infancy. It comprises three areas: parent, child, and interaction. Each area includes different scales: the parent scale includes the subscales “Unfit Parent”, “Intrusive Parent”, and “Detached Parent”; the child scale includes the subscales “Anxious Child” and “Withdrawn Child”; and the interaction scale includes the subscales “Healthy Relationship”, “Dysfunctional Relationship”, “Contingent Problems”, and “Anxious Relationship”.

The first scale refers to parents’ contributions to the relationship with reference to the behavior they engage in with their children, through items such as “The parent is able to fully support the functional capabilities appropriate to the age of the child” (item 5). The second refers to the children’s contributions to the relationship with the parents, through items such as “The child manifests provocative and aggressive behaviors toward the parent” (item 42). Finally, the third scale refers to the overall quality of interactions between the two through items such as “The relationship, even in the absence of conflict, may be inappropriate from the point-of-view of the child’s development” (item 11).

In addition to providing an accurate description of relational patterns, this scale showed an accurate ability to discriminate different relational patterns that may occur in different clinical conditions. To differentiate between clinical and non-clinical conditions the cutoffs have been identified by Quintigliano et al. [10].

### 2.3. Statistical Analyses

The factorial structure of the P-CRS was investigated using confirmatory factor analysis (CFA) as implemented in Jamovi 2.6.19. The models tested for clinician ratings of father–child relationships were based on prior exploratory factor analysis (EFA) of clinician ratings of mother–child relationships [4]. The primary objective of the analysis was to test whether factors emerging from mother–child data would also apply to fathers, thereby assessing whether P-CRS items yield a consistent factor structure across parental roles. According to Speranza et al. [4], three distinct item sets were submitted to CFA separately. These sets were labeled as interaction, parent, and child, each representing specific aspects of the parent–child relationship.

The analysis of the interaction set included 17 items and hypothesized four correlated latent variables: Healthy Relationship (F1, loaded by items 1, 2, 3, and 4, all reverse scored), Dysfunctional Relationship (F2, loaded by items 10, 11, 12, 25, 28, 29, 30, 31, and 37), Contingent Problems (F3, loaded by items 6 and 7), and Anxious Relationship (F4, loaded by items 32 and 36). For the parent set, 13 items were analyzed, with three proposed latent variables. These were as follows: Psychologically Unfit Parent (F1, loaded by items 9, 17, 18, 23, 24, 27, 33, and 5, with this latter reverse scored), Intrusive Parent (F3, loaded by items 15 and 34), and Detached Parent (F3, loaded by items 26, 38, and 45). Lastly, the analysis of the child set involved 6 items, positing two latent factors, labeled Withdrawn Child (F1, loaded by items 13, 19, 20, and 42) and Anxious Child (F2, loaded by items 22 and 35).

Because the clinician ratings were inherently categorical (ranging from 1 = not at all descriptive to 5 = strongly descriptive), the Weighted Least Square Mean and Variance adjusted (WLSMV) estimator was employed for the analyses. This estimator is suitable for ordinal categorical data and does not rely on specific distributional assumptions [36]. For the same reason, scale internal consistency was assessed using the modified Cronbach’s alpha for ordinal items [37].

Each model’s fit was evaluated using the scaled WLSMV χ^2^ statistic and the following fit indices with their corresponding thresholds for excellent fit: Comparative Fit Index (CFI > 0.95), Tucker–Lewis Index (TLI > 0.95), scaled Root Mean Square Error of Approximation (RMSEA < 0.05), and scaled Standardized Root Mean Square Residual (SRMR < 0.05) [38].

Furthermore, the correlations between the obtained factors have been calculated. Cohen [39] established principles for assessing the extent of a correlation and estimating power. Specifically, r = 0.10, r = 0.30, and r = 0.50 were indicated as being small, medium, and large in magnitude, respectively.

Pragmatic considerations primarily determined the sample size in our study due to the difficulty of reaching this population. However, we conducted a post hoc power analysis to determine the statistical power needed to reject a model based on actual and expected RMSEA for a poorly fitting model [40]. Low power (i.e., <0.80) means that if the researcher’s model is false, the probability of detecting this misspecification is low [41]. For the interaction set model, the analysis was based on our current sample size (N = 204) with a calculated RMSEA of 0.076, α level of 0.05, 113 degrees of freedom, and a hypothesized RMSEA of 0.10 (a threshold above which a model is considered unacceptable). The power analyses were performed for the parent and child sets by adjusting the degrees of freedom (62 and 8, respectively) and the corresponding RMSEA values (0.092 and 0.000, respectively). These analyses supported the adequacy of the sample for the interaction and child models, with power values of 0.94 and 1.00, respectively. However, the power computed for the parent model, 0.20, was below the recommended threshold. Therefore, caution should be exercised when evaluating the fit of the parent model.

## 3. Results

### 3.1. Interaction Set

Despite a statistically significant chi-square (χ^2^ = 246.37; df = 113, *p* < 0.001), all the indices supported at least an acceptable model fit. The CFI and TLI, both equal to 0.997, indicated an excellent fit. The RMSEA equal to 0.076 was slightly above the ideal cutoff of 0.05 but remained within an acceptable range (i.e., up to 0.08). Similarly, the SRMR was 0.061, a value within the acceptable range (i.e., up to 0.10).

As shown in Table 2, the Healthy Relationship factor (see F1 in Table 2) was characterized by high factor loadings ranging from 0.57 to 0.70. These items captured positive, reciprocal, and anxiety-free interactions between the parent and the child, reflecting the presence of warmth, mutual pleasure, and adaptive interaction patterns. The Cronbach’s alpha for items expected to load on F1 (α = 0.89) supported its internal consistency and reliability of clinician ratings for individual assessment. The Dysfunctional Relationship factor (see F2 in Table 2) also showed high factor loadings, ranging from 0.78 to 0.95, reflecting patterns of interaction characterized by detachment, emotional disconnection, and dysfunctional interactions. The items loading on this factor also reflected issues such as the lack of vitality, mutual pleasure, and predictability in the relationship. The Cronbach’s alpha for F2 was noteworthy (α = 0.96), highlighting the reliability of clinician ratings associated with this construct. The Contingent Problems factor (see F3 in Table 2) exhibited moderate to strong loadings, ranging from 0.58 to 0.95. This factor captured disturbances in parent–child relationships in specific areas such as negotiation, behavior regulation, and flexibility under stress. While Cronbach’s alpha for F3 was lower (α = 0.71) than F1 and F2, it was still within the acceptable range, suggesting adequate reliability for this factor. Lastly, the Anxious Relationship factor (F4 in Table 2) included items reflecting hyper-responsiveness, tension, and observable anxiety within the parent–child interaction. Factor loadings ranged from 0.75 to 0.90, demonstrating that the selected items strongly represented the hypothesized latent variable. The Cronbach’s alpha for F4 (α = 0.80) indicated good internal consistency.

The correlations between factors provided additional insights into the structure of the “Interaction” area. The Healthy Relationship factor (F1) was strongly correlated with both Dysfunctional Relationship (F2, r = 0.82) and Anxious Relationship (F4, r = 0.79). Considering that the items comprising this factor are reversed, it can be stated that this correlation reflects an inverse relationship between healthy and maladaptive interaction patterns. F2 and F4 exhibited the strongest correlation (r = 0.88), suggesting a high degree of conceptual overlap between these constructs. In contrast, the weaker correlations involving Contingent Problems (F3) indicated that this factor captured unique, situational disturbances that were not as closely tied to the broader patterns of dysfunction or anxiety. The weaker correlations between F3 and the other factors (ranging from 0.37 to 0.51) indicated that this factor represented more specific and situational aspects of the parent–child relationship that were relatively distinct from the broader constructs of dysfunction and anxiety.

### 3.2. Parent Set

Although the model was statistically significant (χ^2^ = 168.07; df = 62, *p* < 0.001), the CFI and TLI, respectively, equal to 0.996 and 0.995, indicated excellent fit. The SRMR equal to 0.072 indicated an acceptable fit, whereas the RMSEA equal to 0.092 was out of the acceptable range. Overall, the fit indices suggested an adequate fit for the model. All the items significantly contributed to their respective factors.

As shown in Table 3, the Psychologically Unfit Parent (F1) factor showed factor loadings between 0.72 and 0.89, reflecting a range of behaviors characterized by insensitivity, unresponsiveness, and psychological inadequacies in parental functioning. Items under this factor captured deficiencies in supporting the child’s functional development and emotional needs. The internal consistency of this factor was excellent, with Cronbach’s alpha of 0.94. The Intrusive Parent (F2) factor was characterized by loadings ranging from 0.59 to 0.96. The highest loading item—“The parent often interferes with the child’s goals and wishes as she/he does not perceive her/him as a separate individual and with her/his own needs” (0.96)—highlighted the overprotective and controlling tendencies captured by this factor. The Cronbach’s alpha for F2 was acceptable (α = 0.721). The relatively low reliability could be due to the heterogeneity in the factor loading size. The Detached Parent (F3) factor included two items, whose loadings were 0.78 to 0.83, respectively. These items captured the father’s emotional detachment and insensitivity to the child’s needs. The items described behaviors such as ignoring or refusing to comfort the child and misinterpreting the child’s signals. The reliability of this factor was good, with a Cronbach’s alpha of 0.88.

The three factors were significantly intercorrelated, underscoring that the three parent constructs were intertwined. For example, F1 (Psychologically Unfit Parent) was highly correlated with both F2 (Intrusive Parent, r = 0.92) and F3 (Detached Parent, r = 0.97), suggesting that a psychologically unfit father could also be intrusive and detached. Similarly, F2 and F3 were strongly correlated (r = 0.83), reflecting some shared features while maintaining their conceptual distinctiveness.

### 3.3. Child Set

Given the relative model simplicity for this item set, the two-factor model was not statistically significant (χ^2^ = 6.63; df = 8, *p* = 0.557), and all fit indices were excellent. For example, the CFI and TLI were equal to 1.000 and 1.005, respectively. Likewise, the RMSEA and SRMR were equal to 0.000 and 0.031. All factor loadings were statistically significant, with values ranging from moderate to high.

As shown in Table 4, for the Withdrawal Child (F1) factor, the loadings ranged from 0.57 to 0.96, indicating that items such as “The child shows a narrow range of affective expressions” and “The child has a disability that alters the parent’s ability to maintain an adequate relationship” (0.918) strongly contributed to defining this construct. Similarly, for the Anxious Child (F2) factor the loadings ranged from 0.51 to 0.88, with the item “The child is condescending or anxious towards the parent in an unusual way” demonstrating the strongest association with this factor. F1 exhibited excellent internal consistency (α = 0.88), while F2 showed only moderate reliability (α = 0.62).

The correlation between the two factors (r = 0.55) indicated a moderate positive relationship, reflecting related but distinct constructs.

## 4. Discussion

The present study aimed to evaluate the utility of an innovative observational tool, called P-CRS, for assessing parent–child relationships in a sample of Italian fathers and their children. While the mother–child relationship has been extensively explored in the academic literature, father–child relationships remain comparatively under-researched. Similarly to the literature on mother–child interactions, the father–child relationship has been insufficiently assessed using clinician-report observational tools, with the majority of studies relying on self-report instruments [10].

Building on previous studies [4,10], we hypothesized that the instrument, which was previously validated with a group of mothers, would retain the same psychometric properties when applied to fathers. To test this, we conducted a confirmatory factor analysis with the fathers’ group based on the exploratory factor analysis previously conducted with mothers [4]. Indeed, the literature highlights both differences and similarities between the maternal and paternal roles, suggesting that in terms of relationship quality, interaction, and the contributions of both parent and child to the interaction, no significant differences emerge based on the parent’s gender or role within the family. The results essentially confirm the hypothesis. All factors within the three categories—“Interaction”, “Parent”, and “Child”—demonstrate at least an adequate fit to the model.

As already pointed out, the P-CRS scale conceptualizes “Interaction” in the parent–child relationship through four key dimensions: Dysfunctional Relationship, Healthy Relationship, Contingent Problems, and Anxious Relationship. This framework not only helps to highlight the eventual resources of the dyad and distinguishes between healthy and dysfunctional aspects of the relationships but also captures nuanced aspects of problematic relationships, such as the anxious quality of some interactions between parents and children, often identified as a potential risk factor for child development [42]. Additionally, the Contingent Problems dimension addresses the adaptability and flexibility of interactions, considered essential qualities of relationships for promoting development [43,44]. Moreover, these dimensions align with established measures of parent–child relationships, such as the PIR-GAS [7] and the RPCL [7], as well as with Anders’s classification of relational patterns, which differentiate between parent–child difficulties in terms of *perturbations*, *disturbances*, and *disorders*, based on how long the impairment lasts and how much it affects different areas of development [45]. As for the “Parent” dimension, three key categories were confirmed: Psychologically Unfit Parent, Intrusive Parent, and Detached Parent. These categories align with other tools present in the literature and are also consistent with established frameworks [4,46]. Similarly, the “Child” dimension is composed of two categories: Withdrawal Child and Anxious Child. These aspects represent two extreme ends of a continuum regarding the child’s involvement in their relationship with a caregiver [4].

These results provide a scale for investigating the role that both mothers and fathers play in early relationship with their children, while also recognizing how fathers may give their specific contribution to development, in both adaptive and maladaptive ways. As other studies have shown, fathers may play a crucial role in children’s development, particularly in areas such as emotional regulation, risk assessment, and cognitive growth through play [35], and in ways that differ from mothers [22]. For example, fathers’ early involvement in play and cognitive activities is linked to better academic outcomes later in childhood, including improved reading and math skills, as well as enhanced self-regulation in the child [35,46]. Additionally, fathers positively influence their children by reducing maternal stress, supporting maternal parenting, and contributing to the family’s financial and emotional stability [35,47].

In addition, a measure that specifically assesses the interaction between parents and their children, with a focus on the child’s specific contribution, also provides an opportunity to assess how the child’s behavior may differ in different relationships, e.g., with their mother or father.

Now that we know the P-CRS can be used with both parents, it would be interesting to explore the differences that may emerge within the parental couple in relation to their children and examine how these varying interactions might evolve over time.

Scientific literature highlights that family dynamics, including interactions with mothers and siblings, shape father–child relationships. For example, supportive co-parenting enhances fathers’ positive involvement with their children as well as variables more closely related to couple functioning, such as marital satisfaction [48]. Additionally, fathers’ work environments and social networks influence the time and energy they can dedicate to parenting. Cultural, political, and economic conditions, such as societal expectations regarding the paternal role, also play a crucial role in shaping fathers’ behavior [35,47]. Therefore, it would be particularly interesting to study how the father’s contribution to the relationship interacts with that of the mother or co-parent, or how it adapts in the case of single fathers. It would also be intriguing to compare this measure with other significant relationships and with the broader environment surrounding the father–child dyad. In this regard, following Belsky’s parenting model [46], it is essential to consider the interplay between multiple factors—contextual, paternal individual characteristics, and children’s individual traits—in shaping healthy child development. These interconnected dimensions emphasize the importance of a holistic approach to understanding and supporting the family dynamics that contribute to children’s well-being.

From a clinical perspective, it would also be beneficial to replicate the methodology used by Quintigliano et al. [10] with father–child dyads to determine whether the P-CRS can similarly differentiate between clinical and non-clinical paternal interactions and identify specific risk factors for the development of affective and relational disorders in children.

In addition to its diagnostic value, the P-CRS could serve as an outcome measure in parent–child dyad therapy by tracking progress throughout the course of treatment. As a clinician-reported tool that focuses on the relationship rather than the symptoms of individual members of the dyad, it allows clinicians to monitor treatment progress without relying on patient self-reports or other potentially invasive tools that could disrupt the therapeutic process. Furthermore, the integration of innovative observational tools alongside traditional self-report questionnaires—whose limitations are well documented—offers a more comprehensive understanding of the case being treated. This approach aligns with the recommendations of the *Diagnostic Classification of Mental Health and Developmental Disorders of Infancy and Early Childhood* (Revised Edition) (DC:0–3R; [7]), DC:0–5 [8], and the *Psychodynamic Diagnostic Manual* (PDM-2; [9]). Given its ability to capture relational aspects linked to various childhood syndromes [10], the P-CRS can also provide valuable insights into the child’s health status during the intervention.

In addition to the strengths highlighted, it is important to acknowledge the study’s limitations. The first concerns the distribution of the sample. Due to challenges in recruiting fathers, it was not possible to achieve a more balanced distribution between, for example, separated/divorced fathers and those still in a couple. In fact, separated/divorced fathers make up more than half of the sample, and this high percentage may seem unusual. A possible explanation is the general tendency for fathers to delegate much of their children’s care—including psychological support—to mothers. However, separated fathers may be more inclined, or even required, to take a more active role in childcare. This is particularly true for children in the clinical group, but it may also apply to children in the non-clinical group. In this case, fathers may have been more willing to engage when they felt the need to demonstrate their ability to care for their children.

Additionally, the children’s group is not homogeneous in terms of age distribution, gender, or diagnosis. These problems can be attributed to the aforementioned issues regarding sample recovery. However, regarding age, all the children were selected within the preschool age range, following the DC:0–5 [8]. While this choice is based on a strong theoretical foundation, it is important to acknowledge that the first five years of life involve rapid developmental changes, which can lead to significant differences between various age groups. Despite this, however, the items in the P-CRS are general and refer to observable behaviors, minimizing the need for inference. They are designed to assess relational and interactive experiences that all the children, regardless of their specific abilities or developmental achievements, may have with their parents.

Furthermore, assessments were conducted by a single observer who, despite being properly trained, may introduce bias or errors. However, as experienced clinicians, the observers are well equipped to evaluate parent–child relationships, which is a key focus of their training in developmental psychology. Moreover, the P-CRS is not strictly an observational tool but rather a guide to various aspects of the relationship that can be observed.

Finally, another limitation of our study is the limited statistical power observed for the parent model, which may hinder our ability to detect potential misspecifications with the current sample size if the proposed structure is inaccurate. It is worth noting, however, that our sample exceeded 200 participants, consistent with accepted confirmatory factor analysis guidelines [41]. In addition, the parent set model demonstrated an acceptable fit. This finding helped to alleviate concerns about the reliability of our findings. Future studies should consider increasing the sample size or adopting alternative methodological strategies (e.g., reducing the model complexity using selected factor markers) to improve the sensitivity for detecting any misspecification in the parent model.

In conclusion, measures of paternal involvement have historically been based on maternal behaviors, limiting their relevance to the unique roles of fathers. As a result, there has been a need to develop better tools and frameworks to capture the specific ways in which fathers interact with their children, and in this regard, the P-CRS makes a valuable contribution. Additionally, future research could extend the application of the P-CRS to diverse family structures, such as single-parent families, same-sex couples, or multicultural families, to explore its versatility and reliability across various contexts.

## Figures and Tables

**Table 1:** Descriptive sample statistics.

Child	N = 204	% (*n*)
Age in months	43.3 (±16.5)	
Gender		
Male	127	62.3
Female	77	37.7
Nationality		
Italian	191	93.6
Others	13	6.4
Diagnosis		
No diagnosis	75	36.8
NOG ^1^	21	10.3
ASD ^2^	28	13.7
DD ^3^	39	19.1
Prematurely	17	8.3
ARD ^4^	14	6.9
Feeding disorder	10	4.9
**Fathers**	**N = 204**	**% (*n*)**
Age	38.4 (±5.9)	
Marital Status		
M/C ^5^	86	42.2
S/D ^6^	118	57.8
Employment Status		
Employee	148	72.5
Self-Employed	51	25
Entrepreneur	4	2
Unemployed	1	0.5

^1^ neurological disease, organic illness, or genetic condition; ^2^ autism spectrum disorder; ^3^ developmental delay; ^4^ affective or relational disorders; ^5^ married/cohabiting; ^6^ separated/divorced.

**Table 2:** Interaction area confirmatory factor analysis and correlation matrix between factors.

Items	F1	F2	F3	F4
1R. The interactions are pleasant for the child and for the parent and without reasons of anxiety	0.793			
2R. The relationship is a stimulus for the growth of both the child and the parent	0.969			
3R. The interactions are reciprocal and synchronous	0.895			
4R. Sometimes the parent and the child may be in conflict, but this does not last more than a few days	0.570			
10. Most interactions between the child and the parent are conflicting and associated with a state of anxiety		0.841		
11. The relationship, even in the absence of conflict, may be inappropriate from the point of view of the child’s development (e.g., the child is treated as younger than his age)		0.888		
12. In the relationship there are dysfunctional patterns that appear deeply rooted		0.952		
25. There is a lack of coherence between the attitudes expressed by the parent towards the child and the observable quality of the interactions (predictability and/or reciprocity are absent in the sequence and order of exchanges)		0.811		
28. Interactions lack vitality and mutual pleasure		0.893		
29. The child and the parent appear detached, with little eye contact and little physical closeness		0.780		
30. The affective tone of the relationship is flat, constricted and characterized by withdrawal and sadness		0.816		
31. Interactions are tense and do not give a sense of tranquility, fun or mutuality		0.893		
37. The relationship is characterized by rough and abrupt interactions, often devoid of emotional reciprocity		0.781		
6. There is a disturbance in the relationship, but limited to only one aspect of functioning (e.g., power supply, play, regulation, etc.)			0.951	
7. If the child and the parent experience anxiety this lasts for a month or more, however the relationship maintains an adaptive flexibility (e.g., through negotiation)			0.580	
32. The parent and the child present an anxious mood observable through motor tension, apprehension, agitation, facial expression, vocalization or language				0.897
36. Both the parent and the child are hyper-responsive to one another				0.748
**Cronbach’s α**	0.891	0.956	0.711	0.803
**Correlations between factors (Pearson’s r)**				
**F1**	1.000			
**F2**	0.822	1.000		
**F3**	0.373	0.438	1.000	
**F4**	0.793	0.880	0.507	1.000

F1 = Dysfunctional Relationship; F2 = Healthy Relationship; F3 = Contingent Problems; F4 = Anxious Relationship.

**Table 3:** Parent area confirmatory factor analysis and correlation matrix between factors.

Items	F1	F2	F3
5R. The parent is able to fully support the functional capabilities appropriate to the age of the child	0.809		
9. The parent is unable to sustain entire areas of the child’s functioning	0.882		
17. The parent dominates the child, who reacts with provocative behavior	0.732		
18. The parent makes requests that are not appropriate to the child’s level of development	0.882		
23. The parent shows sporadic or infrequent involvement or binding	0.720		
24. The parent is insensitive and/or unresponsive to the child’s signals	0.845		
27. The parent is not able to adequately reflect the affective state of the child	0.894		
33. The parent physically manipulates the child in a clumsy way	0.787		
15. The parent often interferes with the child’s goals and wishes as she/he does not perceive her/him as a separate individual and with her/his own needs		0.955	
34. The parent appears to be overprotective and frequently expresses concern for the child’s well-being, behavior or development		0.590	
26. The parent ignores, refuses or is unable to comfort the child			0.814
38. Especially when she/he sees the child as too dependent and demanding, the parent is insensitive to her/his signals			0.832
45. The parent misinterprets the baby’s crying as a deliberate negative reaction to her/him			0.776
**Cronbach’s α**	0.936	0.721	0.837
**Correlations between factors (Pearson’s r)**			
**F1**	1.000		
**F2**	0.923	1.000	
**F3**	0.967	0.831	1.000

F1 = Psychologically Unfit Parent; F2 = Intrusive Parent; F3 = Detached Parent.

**Table 4:** Child area confirmatory factor analysis and correlation matrix between factors.

Items	F1	F2
13. The child has a disability that alters the parent’s ability to maintain an adequate relationship	0.918	
19. In the interaction with the parent the child may appear to be late in motor skills and/or expressive language	0.836	
20. The child shows a narrow range of affective expressions	0.955	
42. The child manifests provocative and aggressive behaviors towards the parent	0.574	
22. The child shows difficulty in separation		0.512
35. The child is condescending or anxious towards the parent in an unusual way		0.876
**Cronbach’s α**	0.884	0.619
**Correlations between factors (Pearson’s r)**		
**F1**	1.000	
**F2**	0.551	1.000

F1 = Withdrawal Child; F2 = Anxious Child.

## Data Availability

The study was not preregistered. The datasets and the additional materials generated during and/or analyzed during the current study are available from the corresponding author upon reasonable request.
